# *Mychonastes* sp. 246 Suppresses Human Pancreatic Cancer Cell Growth via IGFBP3-PI3K-mTOR Signaling

**DOI:** 10.4014/jmb.2211.11010

**Published:** 2022-12-12

**Authors:** Hyun-Jin Jang, Soon Lee, Eunmi Hong, Kyung June Yim, Yong-Soo Choi, Ji Young Jung, Z-Hun Kim

**Affiliations:** 1 Laboratory of Chemical Biology and Genomics, Korea Research Institute of Bioscience and Biotechnology, Daejeon 34141, Republic of Korea; 2 Division of Analytical Science, Korea Basic Science Institute, Daejeon 34133, Republic of Korea; 3 Microbial Research Department, Nakdonggang National Institute of Biological Resources, Sangju-si 37242, Gyeongsangbuk-do, Republic of Korea; 4 Department of Biotechnology, CHA University, Seongnam 13488, Republic of Korea

**Keywords:** *Mychonastes* sp., pancreatic cancer, cell cycle arrest, IGFBP, PI3K, mTOR

## Abstract

Previously, we confirmed that *Mychonastes* sp. 246 methanolic extract (ME) markedly reduced the viability of BxPC-3 human pancreatic cancer cells. However, the underlying mechanism ME remained unclear. Hence, we attempted to elucidate the anticancer effect of ME on BxPC-3 human pancreatic cancer cells. First, we investigated the components of ME and their cytotoxicity in normal cells. Then, we confirmed the G1 phase arrest mediated growth inhibitory effect of ME using a cell counting assay and cell cycle analysis. Moreover, we found that the migration-inhibitory effect of ME using a Transwell migration assay. Through RNA sequencing, Gene Ontology-based network analysis, and western blotting, we explored the intracellular mechanisms of ME in BxPC-3 cells. ME modulated the intracellular energy metabolism-related pathway by altering the mRNA levels of IGFBP3 and PPARGC1A in BxPC-3 cells and reduced PI3K and mTOR phosphorylation by upregulating IGFBP3 and 4E-BP1 expression. Finally, we verified that ME reduced the growth of three-dimensional (3D) pancreatic cancer spheroids. Our study demonstrates that ME suppresses pancreatic cancer proliferation through the IGFBP3-PI3K-mTOR signaling pathway. This is the first study on the anticancer effect of the ME against pancreatic cancer, suggesting therapeutic possibilities and the underlying mechanism of ME action.

## Introduction

Approximately 500,000 people were diagnosed with pancreatic cancer worldwide in 2020 [1]. Since 2020, the number of patients affected has risen by around 1% annually; pancreatic cancer will be the second cause of cancer-related deaths by 2030 [1, 2]. Despite the serious risk to health posed by pancreatic cancer, the median survival period is only 10–12 months, and the 5-year survival rate is 9% [3, 4]. Thus, the development of effective therapeutic agents for pancreatic cancer is a priority.

Patients with pancreatic cancer generally receive chemotherapy with gemcitabine, erlotinib, modified folinic acid, irinotecan, and oxaliplatin (mFOLFIRINOX) [5]. However, these chemotherapeutic agents induce drug resistance and cause severe side effects, including skin rashes, nausea, and vomiting [4, 5]. Therefore, there is an unmet need for safer therapeutic agents for managing pancreatic cancer patients. Over 60% of the drugs—including cytarabine, trabectedin, and lurbinectedin—that inhibit cancer originate from natural sources [6]. However, the components and pharmacological efficacy of many natural products remain to be elucidated [7].

Microalgae are capable of converting solar energy into chemical energy via photo-synthesis. They can produce various bioactive compounds, such as proteins, carotenoids, and vitamins. In addition, their applications have been harnessed for commercial use as sources of pharmacological and medicinal compounds and as nutrient supplements for human consumption [8, 9]. Thus, various microalgae have been studied for their antioxidant, anti-inflammatory, anticancer, anti-diabetes, and anti-bacterial activities [10]. Microalgae are considered a potentially new resource of bioactive compounds because of the discovery of several anticancer compounds in pharmacological research. In our previous report, MEs of four strains of the genus *Mychonastes* demonstrated antioxidant activity and inhibitory effects on cancer cell proliferation. However, since the anticancer effects and their mechanism of action on microalgae are primarily unknown, identifying the anticancer effect of microalgae could provide clues for developing therapeutic agents [11].

Cancer cells mutate growth factor-related signals to maintain an abnormal cell division [12]. Specifically, in the case of pancreatic cancer, 90% of tumors contain the mutated Ras gene, and the expression of its downstream effectors, such as phosphoinositide 3-kinase (PI3K), protein kinase B (Akt), extracellular signal-regulated kinase (ERK), and mammalian target of rapamycin (mTOR), is also upregulated [13, 14]. Thus, various studies have demonstrated that the inhibition of PI3K-Akt-mTOR signaling is a prospective therapeutic strategy for cancer [15-17]. The PI3K-Akt-mTOR pathway modulates cellular survival and proliferation by regulating the intracellular protein synthesis [18, 19]. PI3K is downstream of receptor tyrosine kinases activated by growth signals, including the epithelial growth factor receptor, insulin, and insulin-like growth factor receptor [20, 21]. Akt, a key mediator of PI3K signaling, triggers protein synthesis, proliferation, and survival by activating mTOR signaling [20, 22]. mTOR, including mTOR complex 1 and 2 (mTORC1 and mTORC2, respectively), are highly involved in nutrient sensing, cell metabolism, and aerobic glycolysis [17]. Therefore, finding new drugs targeting the PI3K-Akt-mTOR path-way will be a prospective therapeutic strategy for pancreatic cancer.

In a previous study, we confirmed that methanolic extracts of *Mychonastes* (ME) inhibited the growth of various cancer cells from the pancreas, lungs, and liver [11]. However, the underlying mechanisms and components remain unclear. In the present study, we identified the anti-cancer effect of ME and provide therapeutic possibility of microalgae for pancreatic cancer treatment.

## Materials and Methods

### *Mychonastes* sp. Cell Culture

*Mychonastes* sp. 246 was locally isolated from the Janggyo-ri, Unbong-eup, Namwon-si, Jeollabuk-do and stored in Freshwater Bioresources Culture Collection of Nakdonggang National Institute of Biological Resources (Korea). The microalgal cells were cultivated in BG11 medium containing 1.5 g/l NaNO_3_, 0.04 g/l K_2_HPO_4_, 0.075 g/l MgSO_4_·7H_2_O, 0.036 g/l CaCl_2_·2H_2_O, 0.006 g/l citric acid, 0.006 g/l ferric ammonium citrate, 0.001 g/l ethylene diamine tetraacetic acid, 0.02 g/l Na_2_CO_3_, and 1 mL of trace metal mix A5 solution (2.86 g/l H_3_BO_3_, 1.81 g/l MnCl_2_·4H_2_O, 0.222 g/l NaMoO_4_·2H_2_O, 0.079 g/l CuSO_4_·5H_2_O, and 0.05 g/l CoCl_2_·6H_2_O). Seed cultures were maintained in 0.7-L bubble column photobioreactors (BC-PBRs) containing 0.5 L of fresh medium, incubated at 22 ± 1°C, and irradiated at 100 μE/m^2^/s using 55-W fluorescent lamps with 5% (v/v) continuous CO_2_ bubbling at 0.1 vvm. Cultures were then scaled up to 2-L BC-PBRs under the same conditions as those used for the 0.5-L BC-PBRs. The culture medium was replaced with fresh BG11 medium every 10 d.

### Preparation of ME

*Mychonastes* sp. 246 cells were harvested using Whatman No. 1 filter paper to prepare ME. The filtered cells were washed thrice with distilled water to remove residual ions and cell debris. For freeze-drying, wet biomass was frozen overnight at −80°C and lyophilized using a freeze dryer (FD8512, IlShinBioBase, Korea). Methanol (99.5%, Daejung, Korea) was added to the dry cells (5 g), followed by sonication using a sonicator (Powersonic 410, Hwashin Tech, Korea) for 20 min. The extract was allowed to react at room temperature for 1 day and then filtered (Whatman No. 2, Whatman International, UK). The extract was concentrated using a rotary evaporator (N-2110; Eyela, Japan), and different concentrations (0, 1, 5, 10, and 20 μg/ml) of the extract were prepared using dimethyl sulfoxide (Sigma-Aldrich, USA).

### Quadruple Time-of-Flight (Q-TOF) Mass Spectrometry (MS) and Spectral Interpretation for Sample Qualification

LC separation was performed using an Acquity I-Class UPLC system (Waters, UK) with an Acquity UPLC BEH C18 column (1.7 μm, 2.1×100 mm). The initial column temperature was 40°C. Mobile phase A was water with 0.1% formic acid, and mobile phase B was acetonitrile with 0.1% formic acid. The injection volume was 5 μl, and the flow rate was 10 μl/min. MS detection was performed using an SYNAPT G2-Si system (Waters). The data acquisition mode was the MS^E^. ESI-positive and ESI-negative ionization modes were used in this study. The source and reservation temperatures were set at 120°C and 350°C, respectively. As an external standard, the lock mass compound used was leucine enkephaline (556.2771 positive, 554.2615 negative). The following operation parameters were used: ESI-positive capillary voltage, 3 kV; negative capillary voltage, 2 kV; and cone voltage, 30 V. The collision energies were set as 6 eV ramp (trap) for the low-energy scan and a 20–50-eV ramp (trap) for the high-energy scan. MassLynx 4.1 Waters controlled LC-MS data acquisition. Acquisition data processing was performed using UNIFI1.8 with a traditional medicine library.

### Comparison of ME Quality and Its Composition

Triple quadrupole LC-MS (ESI) analysis was conducted to quantify the composition of ME. Table 1 compares ME quality, as determined using a high-resolution mass spectrometer and traditional medicine library.

### Cell lines and Cell Viability Assay

The human pancreatic cancer cell line BxPC-3 (ATCC CRL-1687) was purchased from the American Type Culture Collection (USA). Roswell Park Memorial Institute (RPMI) medium, fetal bovine serum (FBS), antibiotic–antimycotic solution (100×), 0.25% trypsin-ethylene diamine tetraacetic acid solution, and phosphate-buffered saline (PBS) were procured from Gibco (USA). The cells were cultured in RPMI medium containing 10%(v/v) FBS and 1% (w/v) antibiotic–antimycotic at 37°C in a 5% (v/v) CO_2_ incubator. Human mesenchymal stem cells (hMSCs) were purchased from Lonza (USA) and cultured in StemMACSTM MSC expansion media (Miltenyi Biotec, Germany). Cells (5 × 10^3^ cells/well) were seeded in 96-well plates in a medium containing 5%FBS and 1% antibiotics. After 24h, the medium was replaced with fresh medium containing each ME concentration for 24, 48, and 72 h. At each time point, the number of viable cells was counted under a microscope (Nikon Eclipse TE300; Nikon, Japan).

### Cell Cycle Analysis

BxPC-3 cells (8 × 10^5^ cells/ml) were seeded into a 100-mm dish and starved in a serum-free medium for 24 h. After treatment with ME for indicated times (12 or 24 h), the cells were fixed with 70% ethanol in 1X PBS at -20°C for 3 days. The collected cells were washed twice with PBS and resuspended in PBS containing 100 μg/ml propidium iodide (PI) solution (Sigma-Aldrich) and 50 μg/ml RNase A (Sigma-Aldrich) for 45 min at room temperature. Cell fluorescence was measured using CytoFlex flow cytometry (Beckman Coulter, USA) and analyzed using FlowJo version 10 (Beckman Coulter).

### Transwell Migration Assay

The migration assay was performed using 24-well plate chambers with Transwell inserts with an 8.0-μm pore (BD Bioscience, USA). First, cells (8 × 10^4^ cells/ml) were seeded into the upper chamber with serum-free medium. Next, 1, 5, 10 and 20 μM of ME in 0.8 ml medium, including serum, were added to the lower chamber. After 12–24 h, migrated cells were stained with crystal violet (Sigma-Aldrich) for 10 min. After washing the cells with PBS, the non-migrated cells were removed using a sterilized cotton swap. Finally, the migrated cells were observed and counted under a microscope (Nikon Eclipse TE300; Nikon).

### RNA Sequencing

Total RNA was isolated and underwent quality assessment with Agilent 2000 bioanalyzer (Agilent Technologies, Netherlands). The library was generated with NEBNext Ultra II Directional RNA-Seq Kit (New England Biolabs, UK). Next, the mRNA was isolated, fragmented and synthesized using the Poly(A) RNA Selection Kit (Lexogen, Austria) according to the manufacturer’s instructions. The Illumina indexes 1-12 were used for indexing. The raw sequencing data underwent quality control by FastQC(https://www.bioinformatics.babraham.ac.uk/projects/fastqc/). HiSeq X 10 (Illumina, USA) was utilized for high-throughput paired-end 100 sequencing, and the adapter and low-quality reads (< Q20) were excluded by FASTX_Trimmer ((http://hannonlab.cshl.edu/fastx_toolkit/) and BBMap ((https://sourceforge.net/projects/bbmap). For analysis, the reads were mapped to the reference genome via TopHat [23]and the expression levels were calculated based on the Fragments Per Kilobase Million (FPKM) mapped reads from Cufflinks [24]. Quantile normalization was performed via EdgeR (R Development Core Team, 2016) and ExDEGA (E- biogen, Inc., Korea) was used for mining and graphic visualization of the data. A more detailed description of the method is provided in our previous study [4].

### Gene Ontology-Based Network Analysis

The biological functions of the regulated genes (changing over 2-fold by ME treatment) were determined through an interaction network using the STRING database (http://string-db.org/). The biological functions of the differentially expressed genes and proteins were analyzed according to Gene Ontology-related interaction networks, including cell death- and proliferation-related signaling. Network generation was modulated based on the obtained expression profiles to produce highly connected networks (Network type, full STRING network; meaning of network edges, evidence; minimum required interaction score, highest confidence).

### Immunoblotting

Cell lysates were prepared in RIPA buffer containing protease inhibitor cocktail (Roche Diagnostics, Switzerland). The protein content of the cell lysates was quantified using the Bradford assay [25]. The proteins (30 μg) were resolved using 10–12% sodium dodecyl sulfate-polyacrylamide gel electrophoresis and transferred to a polyvinylidene fluoride membrane (GE Healthcare, USA). The membrane was blocked for 1 h using 5% (w/v) non-fat dry milk in Tris-buffered saline containing Tween-20 and incubated with primary and secondary antibodies according to the manufacturer’s protocol. Antibodies against p-Akt (Ser273), Akt, p-PI3K (Tyr458/Tyr199), PI3K, p-AMPK (Thr172), AMPK, p-mTOR (Ser2448), mTOR, p-p70S6K (Thr389), p70S6K, p-eukaryotic translation initiation factor 4E-binding protein 1; p-4E-BP1 (Thr37/46), 4E-BP1 (Cell Signaling Technology, USA), IGFBP3 (Abcam, UK), and glyceraldehyde-3-phosphate dehydrogenase (GAPDH) (Santa Cruz Biotechnology, USA) were used. An enhanced chemiluminescence system (Thermo Fisher Scientific, USA) was used to visualize the bands using the ChemiDoc MP system (Bio-Rad, USA). Densitometry of the bands was performed using the ImageJ software (National Institutes of Health,USA). Protein levels were quantitatively analyzed and normalized to GAPDH.

### Three-Dimensional (3D) Spheroid Culture

Cells were seeded in 96-well ultra-low attachment microplates (500 cells/well) and cultured after centrifugation at 200 ×*g* for 5 min. Spheroids were allowed to form for three days after seeding before ME treatment. On day 3 of treatment, the medium was replaced with a fresh ME-containing medium. Morphological changes in the spheroids were observed for 12 days, and the volume of the spheroids was evaluated using the NIS-Elements imaging software (Nikon).

### Statistical Analyses

Prism (GraphPad Software, USA) was used for statistical analyses. All measurements were performed at least in duplicate, and all values are expressed as the mean ± standard error of the mean. The results were processed using analysis of variance with Tukey’s test to assess the statistical significance of the differences between groups. Statistical significance was set at *p* < 0.05.

## Results

### Components in ME

**Fig. 1:**
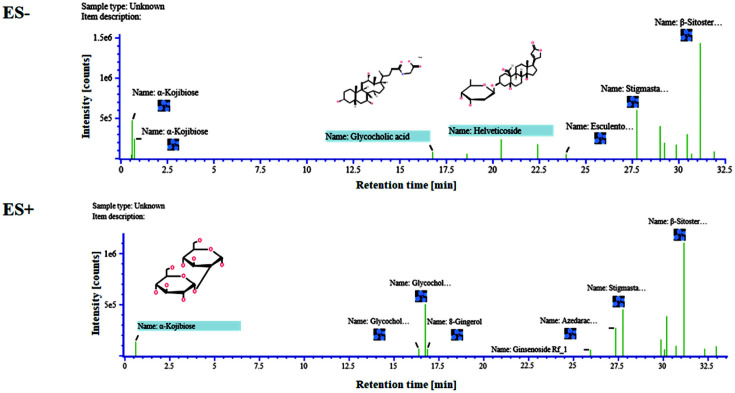
Analysis of components in *Mychonastes* sp. 246 methanolic extracts (ME).

First, to investigate the components of ME, we analyzed the ME using a high-resolution mass spectrometer and a traditional medicine library. Through the investigation of ME on positive electrospray ionization (ES+) and negative electrospray ionization (ES-) TOF MS, we examined both positively and negatively charged components in the extract. As shown in Fig. 1 and Table 1, β-sitosterol tetra-O-acetyl-β-D-glucopyranoside, stigmasta-3α,5α-diol-3-O-β-D-glucopyranoside tetraacetate, and glycocholic acid were highly abundant in ME. In addition, the response values of β-sitosterol tetra-O-acetyl-β-D-glucopyranoside, component 12 in ES- and 18 in ES+, were measured over 2-fold than stigmasta-3α,5α-diol-3-O-β-D-glycopyranoside tetraacetate and glycocholic acid, which are the next most abundant components in ME. Thus, β-sitosterol tetra-O-acetyl-β-d-glucopyranoside may be a significant component of ME.

### ME Treatment Explicitly Suppresses Pancreatic Cancer Cell Growth, Not Normal Cell Growth

**Fig. 2:**
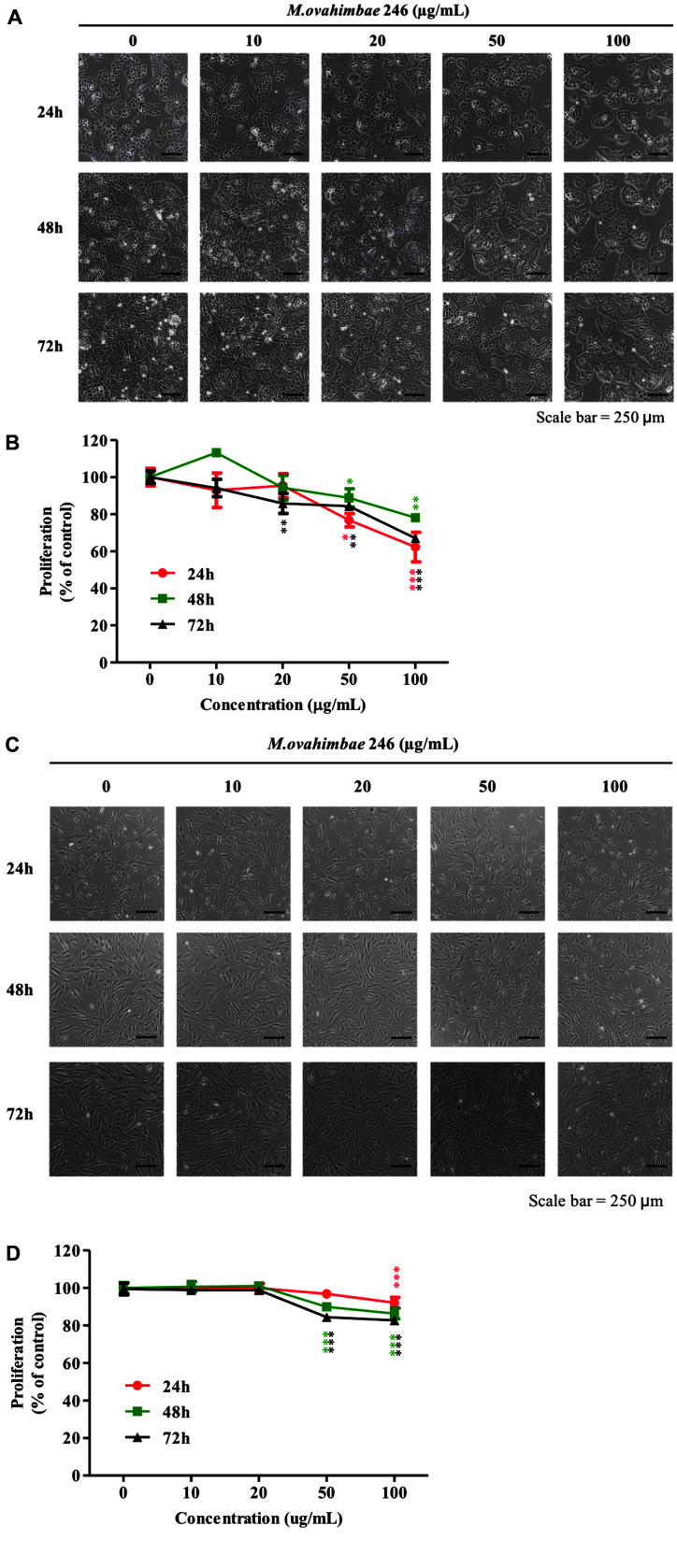
*Mychonastes* sp. 246 methanolic extracts (ME) treatment suppresses proliferation of BxPC-3 pancreatic cancer cells and not human mesenchymal stem cells (hMSC). Morphological changes in pancreatic cancer cells. (**A**) BxPC-3, and (**C**) hMSCs were treated with 10, 20, 50, and 100 μg/ml ME for 24, 48, and 72 h and then observed via microscopy (100× magnification). Growth-inhibitory effect of ME in pancreatic cancer cells. After treatment to ME at indicated concentration for 24, 48 and 72 h, viable (**B**) BxPC-3 cells and (**D**) hMSCs were counted via trypan blue staining using a microscope. Data represent the mean ± SEM of three independent experiments. *p < 0.05, **p < 0.01, and ***p < 0.001 vs. non-treated cells.

Next, to determine the effects of ME on human pancreatic cancer cell proliferation, BxPC-3 and hMSCs were treated with 0, 10, 50, and 100 μg/ml ME for 24, 48, and 72 h. ME treatment inhibited cell proliferation in a dose-dependent manner in pancreatic cancer cells; treatment with 50 μg/ml ME for 24 h blocked cell proliferation by approximately 20% (Figs. 2A and 2B). In addition, exposure to 100 μg/ml of ME for 24 h inhibited the proliferation of BxPC-3 cells up to 40%. In contrast, hMSCs did not present any significant decrease in cell viability in response to 24 h of ME treatment up to a concentration of 50 μg/ml (Figs. 2C and 2D). A statistically significant decrease was observed in 24 h of 100 μg/ml treatment, and 50, 100 μg/ml in longer treatment conditions, with the highest toxic effects appearing at 72 h of 100 μg/ml treatment, reaching 82.7% in cell viability. Overall, the toxicity of ME in hMSCs revealed to be significantly lower than in BxPC-3 cells even in high-dose conditions, remaining over 80%of viability, above which is considered inconsequentially toxic, according to previous research [26].

Morphological changes in both cell lines were observed under a light microscope after treatment with different ME concentrations (0, 10, 20, 50, and 100 μg/ml). Exposure to 100 μg/ml ME for 72 h did not induce marked morphological changes in BxPC-3 cells and hMSCs (Fig. 2B). However, ME treatment markedly reduced the density of BxPC-3 cells.

### ME Treatment Induces Cell Cycle Arrest in Pancreatic Cancer Cells

**Fig. 3:**
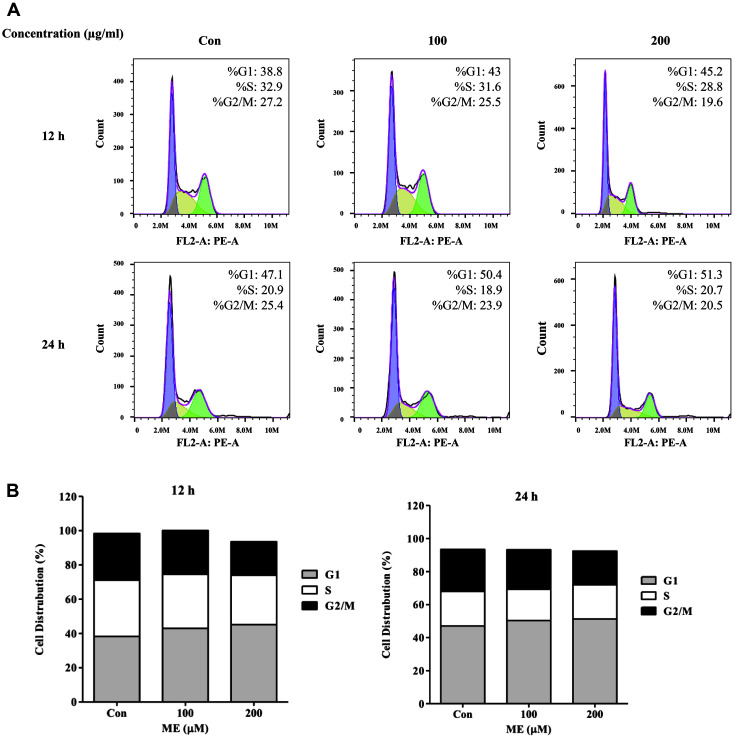
Analysis of cell cycle in BxPC-3 pancreatic cancer cells after treatment with *Mychonastes* sp. 246 methanolic extracts (ME). BxPC-3 cells after staining with propidium iodide fluorescent dyes were evaluated using CytoFlex flow cytometry (Beckman coulter) and FlowJo version 10 (Beckman coulter). Values are expressed as a percentage of cell cycle phases G1, S, and G2/M.

Fig. 2 shows that ME treatment did not induce distinct morphological changes, such as shrunken or buoyant cells. Since dead or dying cells switch their morphology, including shrinking, swelling, and floating [4, 27], we postulated that ME treatment disturbed pancreatic cancer cell growth and did not trigger cancer cell death. Thus, to investigate how ME treatment modulated cancer cell growth, we performed PI staining and flow cytometric analysis of BxPC-3 cells treated with ME (0, 100, and 200 μg/ml) for 12 and 24 h (Fig. 3). ME treatment (200 μg/ml) decreased relative fraction of cells in the G2/M phase 27.2%–19.6% at 12 h and 25.4%–20.5% at 24 h. The proportion of cells in the S phase was reduced by approximately 32.9%–28.8% after 12 h by 200 μg/ml ME. Contrary to the results seen with the G2/M and S phases, ME treatment (200 μg/ml) increased the proportion of cells in the G1 phase from 38.8% to 45.2% at 12 h and from 47.1% to 51.3% at 24 h. Next, we confirmed the regulation of cell cycle-related protein levels by ME treatment (Fig. S1). Treatment with 200 μg/ml ME for 12 h induced the expression of tumor suppressor genes such as p16 and p21, which regulate cell cycle progression. In addition, G1 checkpoint-related protein pRB was upregulated over 2.5-fold by ME treatment in BxPC-3 cells, and expression of cyclin D1 slightly fluctuated. These data suggest that ME triggers G1 phase arrest following S and G2/M phase reduction in BxPC-3 pancreatic cancer cells.

### *Mychonastes* sp. Treatment Inhibits Migration of Pancreatic Cancer Cells

**Fig. 4:**
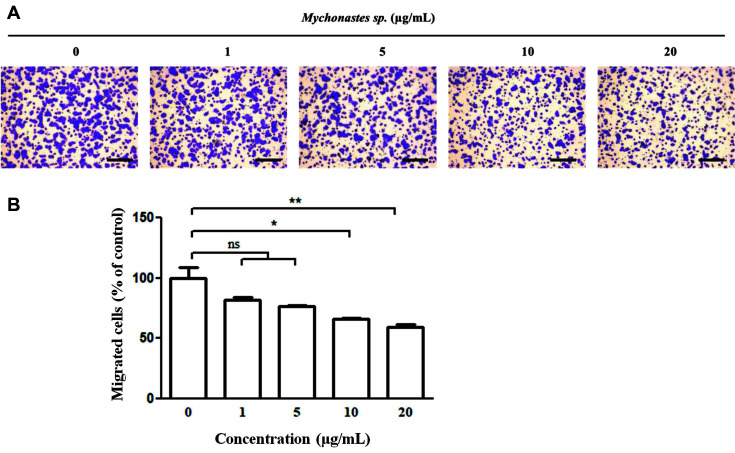
Migration-inhibitory effect of *Mychonastes* sp. 246 methanolic extracts (ME) in BxPC-3 pancreatic cancer cells. (**A**) Migrated cells were examined using a Transwell migration system and visualized after crystal violet staining. (**B**) Data represent the mean ± SEM of three independent experiments. *p < 0.05, and **p < 0.01 vs. control cells.

Pancreatic cancer is difficult to detect in its early stages, and most patients have metastasis at the time of diagnosis [28]. Furthermore, pancreatic cancer is highly aggressive with high metastatic potential [29]. Thus, we performed a Transwell migration assay with ME (0, 1, 5, 10, and 20 μg/ml) to determine the migration-inhibitory effect of ME in pancreatic cancer cells for 16 h (Fig. 4). ME treatment at 20 μg/ml did not markedly affect cancer cell viability. However, the cancer cell migration was blocked by up to 50%. These results suggested that ME even at low toxic concentrations can efficiently block cancer cell migration.

### ME Treatment Modulates the Gene Expression of Human Pancreatic Cancer Cells

**Fig. 5:**
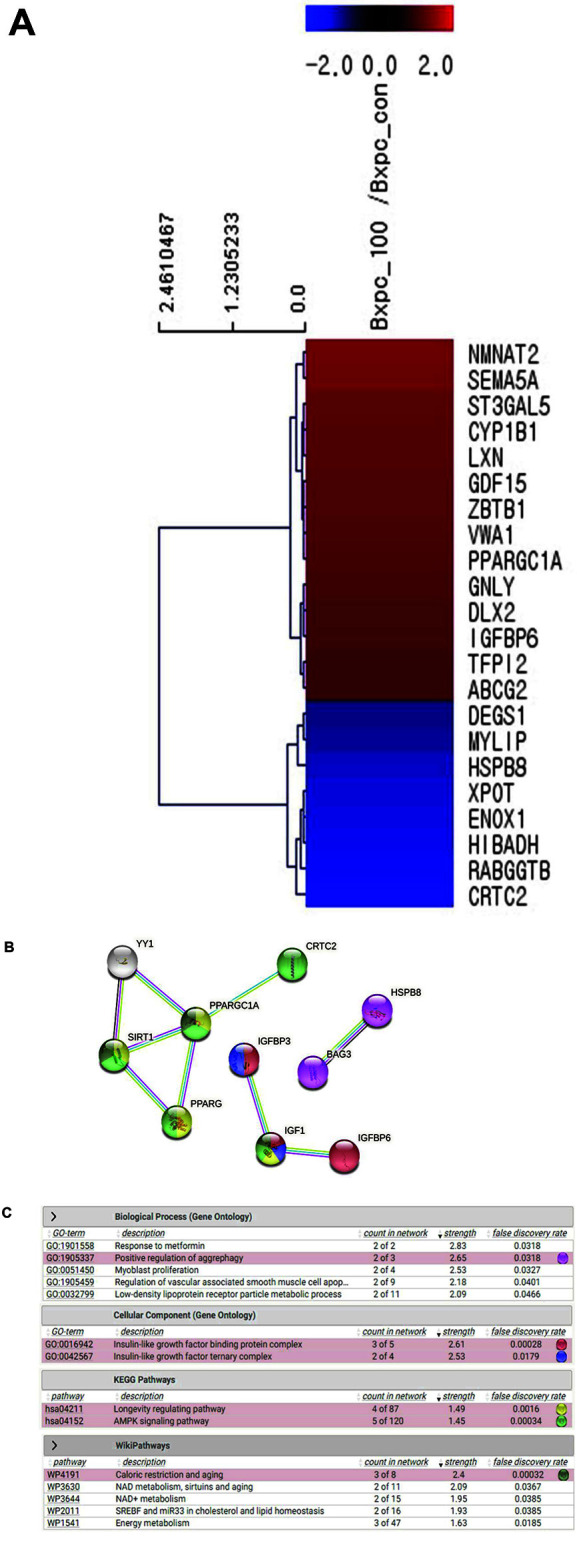
RNA sequencing analysis of the changes in gene expression and signaling network in *Mychonastes* sp. 246 methanolic extracts (ME)-treated BxPC-3 pancreatic cancer cells. Red and blue colors represent genes that were up- or down-regulated, respectively by more than 2-fold. The ratios of gene profiles are presented as a (**A**) heat map and gene expression pattern. (**B**) Image from the STRING website showing results obtained upon analyzing a set of 10 proteins suspected to be involved in the cell death process (GO analysis). The insets show the additional information available for a single protein, a reported enrichment of functional connections among the set of proteins, and statistical enrichments detected in functional subsystems. (**C**) Gene On-tology and Kyoto Encyclopedia of Genes and Genomes and Wiki pathway analysis of protein–protein interactions. Enriched functions were selected and the corresponding protein nodes in the network were automatically highlighted.

Figs. 3 and 4 show that ME treatment blocked cell cycle progression and migration in pancreatic cancer cells. To explore the intracellular pathway of ME, we analyzed gene expression levels through RNA sequencing of BxPC-3 cells treated with 100 μg/ml ME for 24 h. Among the 25,737 unique genes evaluated, the expression levels of 30 genes were changed by ME treatment, among which 22 were upregulated and 8 were downregulated. Using the ExDEGA program [30], we combined the biological features of genes and categorized the genes that play a crucial role in cancer cell proliferation. The cell proliferation-related genes regulated by 2-fold in ME-treated BxPC-3 cells were assessed using the Multiple Experiment Viewer (MeV) tool and hierarchical cluster analysis (Fig. 5). We confirmed that ME treatment modulated the expression of 22 genes, among which 8 genes were downregulated, while 14 genes were upregulated. Furthermore, ME treatment blocked cellular growth by regulating insulin-like growth factor-binding protein 6 (IGFBP6) (Fig. 5A). In addition, the expression of peroxisome proliferator-activated receptor gamma coactivator 1-alpha (PPARGC1A, also known as PGC-1α) and CREB-regulated transcription coactivator 2 (CRTC2) was modulated in ME-treated BxPC-3 cells.

With 2-fold up- or down-regulated genes by ME treatment, we examined the protein–protein interactions and Gene Ontology terms using the STRING database (Figs. 5B and 5C). ME treatment modulated cell proliferation by regulating the insulin-like growth factor-binding protein complex (GO: 0016942; false discovery rate *p* = 0.00028: IGFBP3, IGF1, and IGFBP6), AMPK signaling pathway (hsa04152; false discovery rate *p* = 0.00034: SIRT1, PPARG, PPARGC1A, and CRTC2), caloric restriction, and aging pathways (WP4191; false discovery rate *p* = 0.00032: SIRT1, PPARGC1A, and IGF-1).

### *Mychonastes* sp. Treatment Inhibits mTOR Activation via IGFBP3-PI3K Pathway

**Fig. 6:**
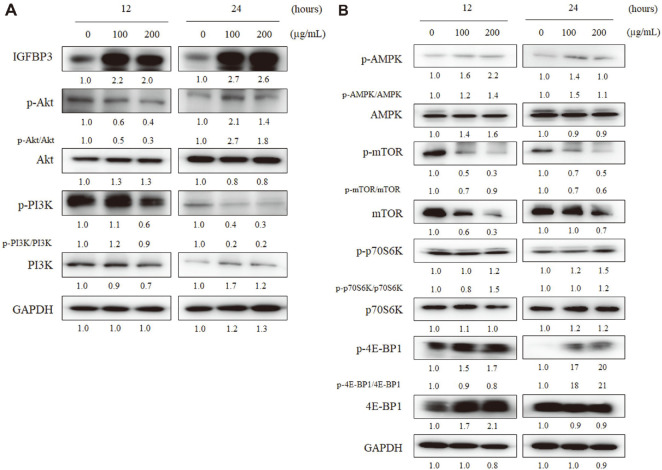
*Mychonastes* sp. 246 methanolic extract (ME) treatment reduced PI3K-mTOR activation with IGFBP3 induction in BxPC-3 pancreatic cancer cells. BxPC-3 cells were treated with ME (0, 100, and 200 μg/ml) for 12 or 24 h and analyzed using western blotting with antibodies against (**A**) IGFBP3, p-PI3K (Tyr458/Tyr199), PI3K, p-Akt (Ser273), Akt, (**B**) p-AMPK (Thr172), AMPK, p-mTOR (Ser2448), mTOR, p-p70S6K (Thr389), p70S6K, p-4E-BP1 (Thr37/ 46) and 4E-BP1. The band densities were quantified and normalized to GAPDH.

As shown in Fig. 5, we confirmed that ME treatment of BxPC-3 cells changed the mRNA levels of IGFBP and AMPK and caloric-related pathways. Based on STRING analysis, we investigated the changes in the intracellular energy-related pathway in ME-treated BxPC-3 cells using western blotting. ME treatment at 100 or 200 μg/ml for 24 h of BxPC-3 cells declined the activation of PI3K and mTOR, and not of Akt, IGFBP3, and 4E-BP1 (Fig. 6). ME treatment at 200 μg/ml for 24 h markedly reduced phosphorylation of PI3K by 0.2-fold and mTOR by 0.6-fold. However, ME treatment induced IGFBP3 expression up to 2.6-fold. In addition, phosphorylation of Akt and 4E-BP1 increased by 1.8-fold and 21-fold, respectively. The activation of AMPK and ribosomal protein S6 kinase beta-1 (S6K1; p70S6K) fluctuated following ME treatment. Thus, ME treatment may inhibit pancreatic cancer cell growth via the IGFBP3-PI3K-mTOR pathway.

### ME Treatment Suppresses the Growth of 3D Spheroidal BxPC-3 Cells

**Fig. 7:**
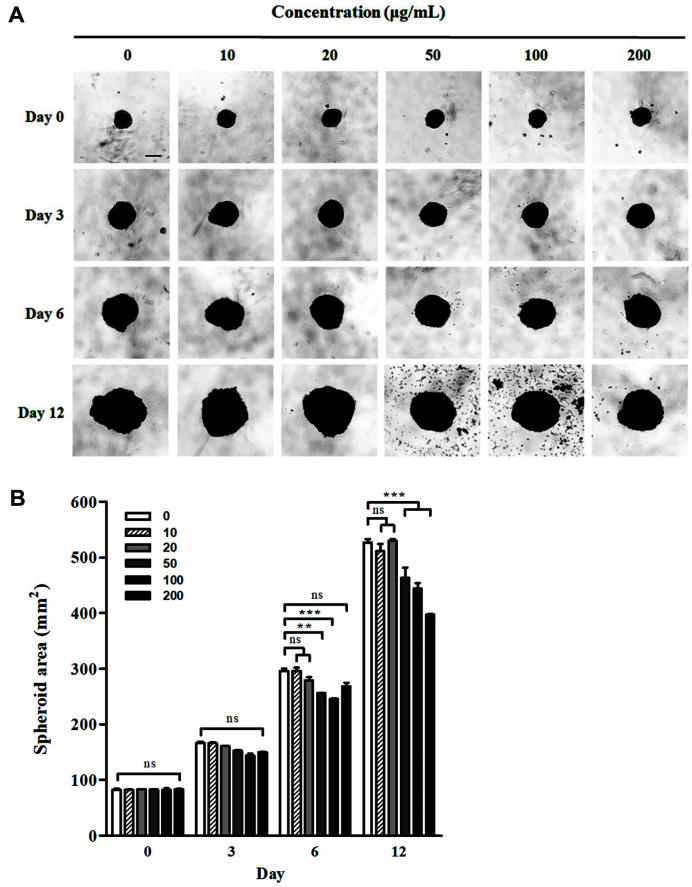
*Mychonastes* sp. 246 methanolic extract (ME) inhibits growth of three-dimensional cultured BxPC-3 spheroids. Cells were cultured for 3 days post-seeding to allow spheroid formation be-fore drug treatment. (**A**) Representative microscopic images of spheroids on day 0, 3, and 6 of treatment. Scale bar represents 250 μm. (**B**) Diameter analysis of spheroids on day 0, 3, 6 and 12 of treatment. Data represent the mean ± standard error of three independent experiments. **p < 0.01 and ***p < 0.0001 vs. vehicle cells.

Although tumors in the body grow in a 3D environment, cancer cells in vitro were cultured in a 2D environment. To compensate for this drawback, we evaluated whether ME treatment decreased the volume of 3D spheroidal BxPC-3 cells. On the third day after seeding, the spheroids were treated with each ME concentration (day 0; Fig. 7). The volume of BxPC-3 spheroids increased for 12 days, but treatment with 50, 100, and 200 ME μg/ml reduced the size of BxPC-3 spheroids (Fig. 7A). With these changes in sphere size, ME treatment (50, 100, and 200 μg/ml) for 12 days triggered a more compact and regular shape of BxPC-3 spheroidal cells by reducing their proliferation rate. While 100 and 200 μg/ml of ME treatment for three days only slightly inhibited the growth of BxPC-3, treatment for day 12 effectively reduced 20% and 25% of the spheroid area, respectively (Fig. 7B). Thus, these data suggest that ME could suppress the growth of 3D-cultured cancer spheroids and 2D cultured cells.

## Discussion

Cancer cells continuously require many nutrients, including oxygen, glucose, amino acids, and lipids, for uninhibited proliferation [28, 31]. Therefore, cancer cells alter their energy metabolism pathways to support the massive demand for nutrients by inducing anaerobic glycolysis and recycling intracellular organelles [31, 32]. Although intracellular metabolic reprogramming is a general feature of cancers, each has a different metabolic alteration strategy suitable for its tissue, genetic mutation, or microenvironment [28, 33]. In pancreatic cancer, metabolic reprogramming promotes cancer progression and metastasis and disrupts therapeutic efficacy [28]. Therefore, regulating metabolic pathways to block cancer progression is a promising therapeutic strategy for successfully managing pancreatic cancer. In this regard, we focused on the inhibitory proliferation effect of ME on BxPC-3 human pancreatic cancer cells via regulation of their energy metabolism.

The genus *Mychonastes*, a freshwater microalga, produces lipids and carotenoids [34, 35]. Although recent studies have revealed the bioactivity of the extracts obtained from the *Mychonastes* genus, such as their antioxidant, anti-fungal, and anticancer effects, its intracellular pathway has not been elucidated [11, 36].

Through GC-MS/MS analysis to investigate the chemical components in ME, we found that β-sitosterol tetra-O-acetyl-β-D-glycopyranoside (C_43_H_68_O_10_) was the most abundant component of the ME (Fig. 1, Table 1). β-Sitosteryl is a phytosterol, a naturally occurring compound structurally like cholesterol [37]. Recent studies have investigated phytosterols’ anticancer effects through cell cycle arrest, apoptosis induction, and blocking angiogenesis [38]. For example, Xu *et al*. revealed that β-Sitosterol-D-glucoside promoted apoptosis of breast cancer via microRNA-10a regulation of the PI3K-Akt pathway [38]. Furthermore, according to a study by Swadesh *et al*., β-Sitosterol-3-O-β-D-glucoside from *Azadirachta indica* triggered apoptosis via DNA fragmentation-mediated G0/G1 arrest in leukemic cells [39]. Moreover, stigmasta-3α,5α–diol 3-O-β-D-glucopyranoside and glycocholic acid, the next most abundant components in ME, were also related to steroids from plants and lipid emulsification [40]. Therefore, we can deduce that abundant β-sitosterol in ME may inhibit pancreatic cancer growth.

In addition to β-sitosterol, microalgal carotenoids or pigments, such as astaxanthin, beta-carotene, and phycoerythrin, have been studied for inducing cell cycle G0/G1 phase arrest in cancer cells [41, 42]. Accordingly, even a small amount of various ME components might comprehensively inhibit cell cycle progression in BxPC-3 cells.

As shown in Fig. 4, ME treatment strikingly inhibited the migration of BxPC-3 cells. Cytoskeleton remodeling is necessary during cell cycle progression and migration [43]. The PI3K signaling pathway regulates cytoskeleton rearrangement, including actin polymerization, an abundance of intermediate filaments, and microtubule stability [44, 45]. The reduced PI3K activation by ME treatment may suppress pancreatic cancer migration (Figs. 4–6). The migration-inhibitory effect of ME could increase its therapeutic efficacy, in hand with its growth-inhibitory effect on pancreatic cancer cells.

On the pancreatic cancer surface, insulin-like growth factor (IGF) and IGF receptors are highly expressed to promote cancer progression [45]. Binding of IGF to the IGF receptor activates PI3K/Akt and ERK signaling leading to cancer cell survival, proliferation, and migration [46]. Conversely, IGFBP suppresses IGF-related signaling pathways by interrupting the IGF-to-IGF receptor binding [47, 48]. In our study, we found that ME treatment upregulated the expression of IGFBP3, the most abundant IGFBP, and downregulated the activation of the PI3K-mTOR signaling pathway (Figs. 5 and 6). According to a study by Kerr *et al*., microRNA-10a, levels of which are increased by β-sitosterol, acts through the IGF- and IGFBP-related pathways in cancer [48]. This suggests that β-sitosterol in ME may play a crucial role in modulating the IGFBP-PI3K pathway in pancreatic cancer cells.

However, ME treatment induced Akt phosphorylation and inactivated PI3K and mTOR (Fig. 6B). Lakshmipathi *et al*. reported that Akt activation might affect the intracellular energy environment [49]. In a metabolic inhibitory situation, downregulated mTOR activates Akt through negative feedback [49]. Akt activation might be more sensitively induced because mTOR is highly activated in pancreatic cancer cells [17]. This suggests that IGFBP3-mediated growth-inhibitory signals may cause the upregulation of Akt phosphorylation in ME-treated cancer cells.

AMPK is a crucial regulator of intracellular energy homeostasis and interacts with the mTOR signaling [49]. AMPK activation regulates protein synthesis through mTOR signaling-mediated p70S6K and 4EBP1 modulation [50, 51]. We also determined the expression of AMPK, a crucial pathway, and mTOR downstream effector of PI3K-Akt signaling based on STRING analysis. ME treatment triggered 4E-BP1 activation and mTOR inactivation, whereas the activation of AMPK and p70S6K was slightly altered (Fig. 6). According to Sook *et al*., β-sitosterol induces ROS-mediated AMPK and MAPK activation [52]. Therefore, the slight increase in AMPK activation by ME treatment might be due to the inactivation of PI3K and activation of Akt and β-sitosterol in ME (Fig. 6A). However, β-Sitosterol is also known as its antioxidant activity [53]. Thus, correlation ROS and ME is needed to be clarified.

mTOR, a master regulator of energy homeostasis, plays a crucial role in coordinating cancer cell proliferation and growth [54, 55]. In particular, the inactivation of mTOR can induce cell cycle G1 phase arrest [19, 54]. 4E-BP1 and p70S6K, modulated by mTOR, are significant mediators of the mTOR-dependent G1 arrest [19, 54]. As shown in Figs. 3 and 6, we confirmed that ME treatment markedly suppressed the activation of mTOR and 4E-BP1 with G1 phase arrest. In contrast to p70S6K, 4E-BP1 directly affects cell cycle regulation via the CAP-dependent mRNA translation [56]. This may correlate with the significant increase in 4E-BP1 activation in ME-treated BxPC-3 cells (Fig. 6B). The regulation of mTOR or AMPK by microalgal extracts has been reported previously, but this study is the first to show that ME treatment changed the 4E-BP1 and p70S6K activation [41, 57, 58].

Like ME, Spirulina microalgae extracts have also been reported to have growth-inhibitory effects on pancreatic cancer cells [59, 60]. Phycocyanin, a pigment-protein in Spirulina extracts, functions as an anticancer compound that regulates PI3K/AKT/mTOR or AMPK signaling [59, 61]. Thus, analyzing chemical components in microalgal extracts is essential to clarify the correlation between extracts and their effects. This study provides a critical clue for the anticancer effect of ME, from its chemical composition to the intracellular mechanism of human pancreatic cancer cells.

A two-dimensional culture system cannot represent the cellular microenvironment in our body; hence, a 3D cell culture model that is closer in vivo, with limited nutrient supply, polarity, and cell–cell interaction, is in the limelight in the fields of drug discovery and drug repositioning [62]. Therefore, it is crucial to investigate drug efficacy using 3D cultured cancer cells, as it can provide accurate data for in vivo trials [63]. As shown in Fig. 7, ME treatment reduced the growth and irregularity of 3D cultured BxPC-3 spheroids. Since the loosened shape of spheroids is caused by low expression of E-cadherin, downregulation of E-cadherin expression could trigger cancer malignancy, including invasion, migration, and proliferation [64, 65]. Hence, ME treatment may hamper the progression of pancreatic tumors, as with 3D cultured cancer spheroids. These data indicate the high application potential of ME in the treatment of human pancreatic cancer.

In anticancer studies, reducing the side effects caused by killing normal cells is a major clinical issue [66]. In particular, rapidly growing normal cells such as bone marrow, germline, and hair papilla cells are vulnerable to chemotherapeutic agents [67]. Our study confirmed that ME treatment was not highly toxic to bone marrow-derived hMSC (Fig. 2). Furthermore, since mTOR levels are markedly higher in pancreatic cancer cells than in normal cells, ME may sensitively act on pancreatic cancer cells than on normal cells. These results show that ME is a prospective anticancer agent with few side effects.

**Fig. 8:**
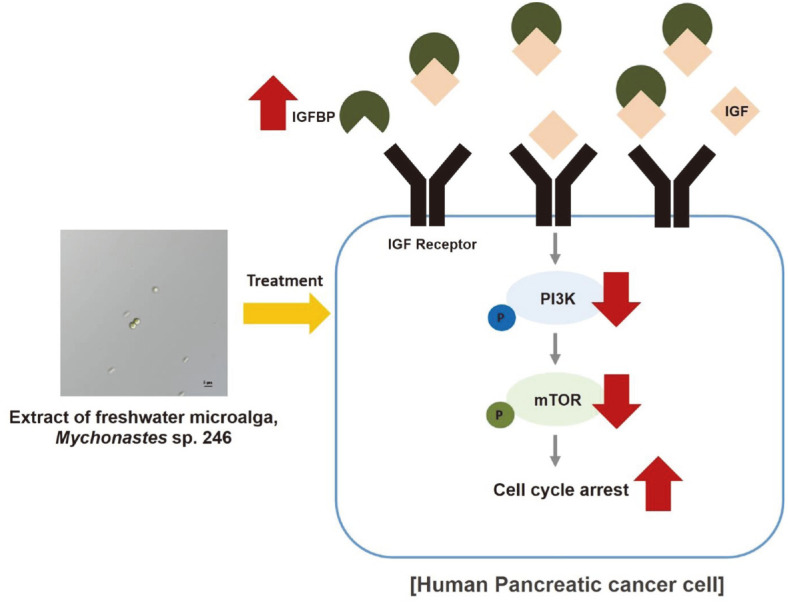
Schema of the growth-inhibitory effects of *Mychonastes* sp. 246 methanolic extracts on human pancreatic cancer cells.

Our study clarified that ME treatment increased cell cycle G1 phase arrest in pancreatic cancer cells for the first time by disrupting energy homeostasis via the IGFBP-PI3K-mTOR signaling pathway (Fig. 8). Additionally, our findings suggest that ME treatment could block the growth of both 2D and 3D cultured pancreatic cancer cells without toxicity to normal cells. In summary, ME represents a potential biosource for anticancer drug development.

## Supplemental Materials

Supplementary data for this paper are available on-line only at http://jmb.or.kr.

## Figures and Tables

**Table 1:** Quantification of *Mychonastes* sp. 246 methanolic extracts (ME) compositions, using highresolution mass spectrometer and traditional medicine library.

No.	Electrospray	Component name	Formula	RT	Response	Observed m/z	Error	Adducts
1	ES-	α-Kojibiose	C12H22O11	0.59	139291	365.1047	-0.6898	+Na, +H
2	ES-	Glycocholic acid	C26H43NO6	16.35	70106	466.3152	-1.1188	+H, +Na
3	ES-	Glycocholic acid	C26H43NO6	16.74	505664	466.3151	-1.165	+H, +Na
4	ES-	8-Gingerol	C19H30O4	16.86	67292	323.2208	-0.8497	+H
5	ES-	Ginsenoside Rf_1	C42H72O14	25.93	60433	801.5008	1.2799	+H
6	ES-	Azedarachin C	C32H42O10	27.36	273606	609.2698	2.802	+Na
7	ES-	Stigmasta-3α,5α-diol-3-O-β-D-glucopyranoside tetracetate	C43H70O11	27.73	455848	785.4803	-0.7287	+Na
8	ES-	Oligomycin	C45H74O11	29.88	160211	813.5112	-1.1658	+Na
9	ES-	Phosphatidyl ethanolamines	C4^1^H80NO8	30.06	63760	768.5531	1.7049	+Na
10	ES-	Acanthoside K2	C43H72O11	30.18	388269	787.4964	-0.2867	+Na, +H
11	ES-	Luteoxanthin	C40H56O4	30.7	98356	601.4236	-1.5808	+H
12	ES-	β-Sitosteryl tetra-O-acetyl-β-D-glycopyranoside	C43H68O10	31.15	1108814	767.4698	-0.6804	+Na
13	ES-	12-Hydroxyhydromethyl abietate	C2^1^H36O3	32.3	68981	337.2727	-0.9909	+H
14	ES-	Soyacerebroside᧓	C4^1^H77NO9	32.95	93281	750.5525	3.405	+Na
15	ES+	Chelidimerine	C43H32N_2_O9	0.57	47837	719.2025	-0.9556	-H
16	ES+	Tribulusamide B	C36H34N_2_O9	0.59	196246	683.2256	0.9933	+HCOO
17	ES+	α-Kojibiose	C12H22O11	0.6	477154	341.1088	-0.1783	-H, +HCOO
18	ES+	Tribulusamide B	C36H34N_2_O9	0.73	91525	683.2253	0.6867	+HCOO
19	ES+	α-Kojibiose	C12H22O11	0.73	245487	341.1086	-0.3234	-H, +HCOO
20	ES+	Glycocholic acid	C26H43NO6	16.75	84875	510.3069	-0.3366	+HCOO
21	ES+	Vitexilactone	C22H34O5	16.75	62308	423.238	-0.8209	+HCOO
22	ES+	Vitetrifolin D	C24H38O5	18.61	59497	451.2685	-1.6288	+HCOO
23	ES+	Helveticoside	C29H42O9	20.46	238288	579.2837	2.6197	+HCOO
24	ES+	Ephedradine C	C30H40N4O5	22.42	178066	581.2981	0.0324	+HCOO
25	ES+	Esculentoside P	C36H56O12	23.96	57410	725.3778	2.4321	+HCOO
26	ES+	Stigmasta-3α,5α-diol-3-O-β-D-glycopyranoside tetracetate	C43H70O11	27.73	605590	807.4901	0.1138	+HCOO, -H
27	ES+	Acanthoside K2	C43H70O11	29.02	403120	809.5049	-0.7577	+HCOO, -H
28	ES+	Oligomycin	C45H74O11	29.23	196651	835.521	-0.3506	+HCOO, -H
29	ES+	Oligomycin	C45H74O11	29.87	174487	835.5209	-0.435	+HCOO, -H
30	ES+	Acanthoside K2	C43H72O11	30.47	303264	809.5053	-0.3424	+HCOO, -H
31	ES+	Saikosaponin E	C42H68O12	30.69	59920	763.4667	2.8627	-H
32	ES+	β-Sitosteryl tetra-O-acetyl-β-D-glucopyranoside	C43H68O10	31.15	1435870	789.4797	0.2224	+HCOO, -H
33	ES+	Acanthoside K3	C42H70O12	31.92	87131	765.4791	-0.3754	-H
