# Supplementary material for: Mychonastes sp. 246 Suppresses Human Pancreatic Cancer Cell Growth via IGFBP3-PI3K-mTOR Signaling
Source: J Microbiol Biotechnol. 2022 Dec 12;33(4):449–62. doi: 10.4014/jmb.2211.11010 (PMC10164724; doi:10.4014/jmb.2211.11010)
Supplement: Supplementary file 1 [file jmb-33-4-449-supple.pdf]

J. Microbiol. Biotechnol.
https://doi.org/10.4014/jmb.2211.11010

Supplementary Data

Mychonastes sp. 246 Suppresses Human Pancreatic Cancer Cell Growth via IGFBP3-PI3K-mTOR Signaling

Hyun-Jin Jang[1], Soon Lee[2], Eunmi Hong[2], Kyung June Yim[3], Yong-Soo Choi[4], Ji Young Jung[3] and Z-Hun Kim[3*#]

[1]Laboratory of Chemical Biology and Genomics, Korea Research Institute of Bioscience and Biotechnology, Daejeon 34141, Republic of Korea

[2]Division of Analytical Science, Korea Basic Science Institute, 169-148 Gwahakro, Daejeon 34133, Republic of Korea

[3]Microbial Research Department, Nakdonggang National Institute of Biological Resources, Sangju-si 37242, Gyeongsangbuk-do, Republic of Korea

[4]Department of Biotechnology, CHA University, Seongnam 13488 Republic of Korea

***Correspondence to:** Dr. Z-Hun Kim (E-mail: kimzhun@nnibr.re.kr,
Microbial Research Department, Nakdonggang National Institute of Biological Resources, Tel: +82-54-530-0841, Fax: +82-54-530-0849)

#Present address: Biojarlam, Huevergreenpharm Inc., Incheon 21447, Republic of Korea

Materials and Methods

Western Blotting

The proteins (30 μg) were resolved by 10-12% sodium dodecyl sulphate-polyacrylamide gel electrophoresis and transferred to a polyvinylidene fluoride membranes. The membrane was blocked for 1 h using 5% (*w/v*) nonfat dry milk in Tris-buffered saline containing Tween-20 (TBST) and incubated with primary and secondary antibodies according to the manufacturer's protocol. Antibodies against Cyclin D1, p-Rb, and Rb(Cell Signaling Technology, USA), p16/ink4a (Abcam, Cambridge, UK), and p21 and GAPDH (Santa Cruz Biotechnology, USA). An enhanced chemiluminescence system (Thermo Fisher Scientific, USA) was used to visualize the bands using the ChemiDoc MP system (Bio-Rad, Hercules, CA, USA). Densitometry of the bands was performed using ImageJ software (National Institutes of Health, USA). Protein levels were quantitatively analyzed and normalized against GAPDH.

Figure S1

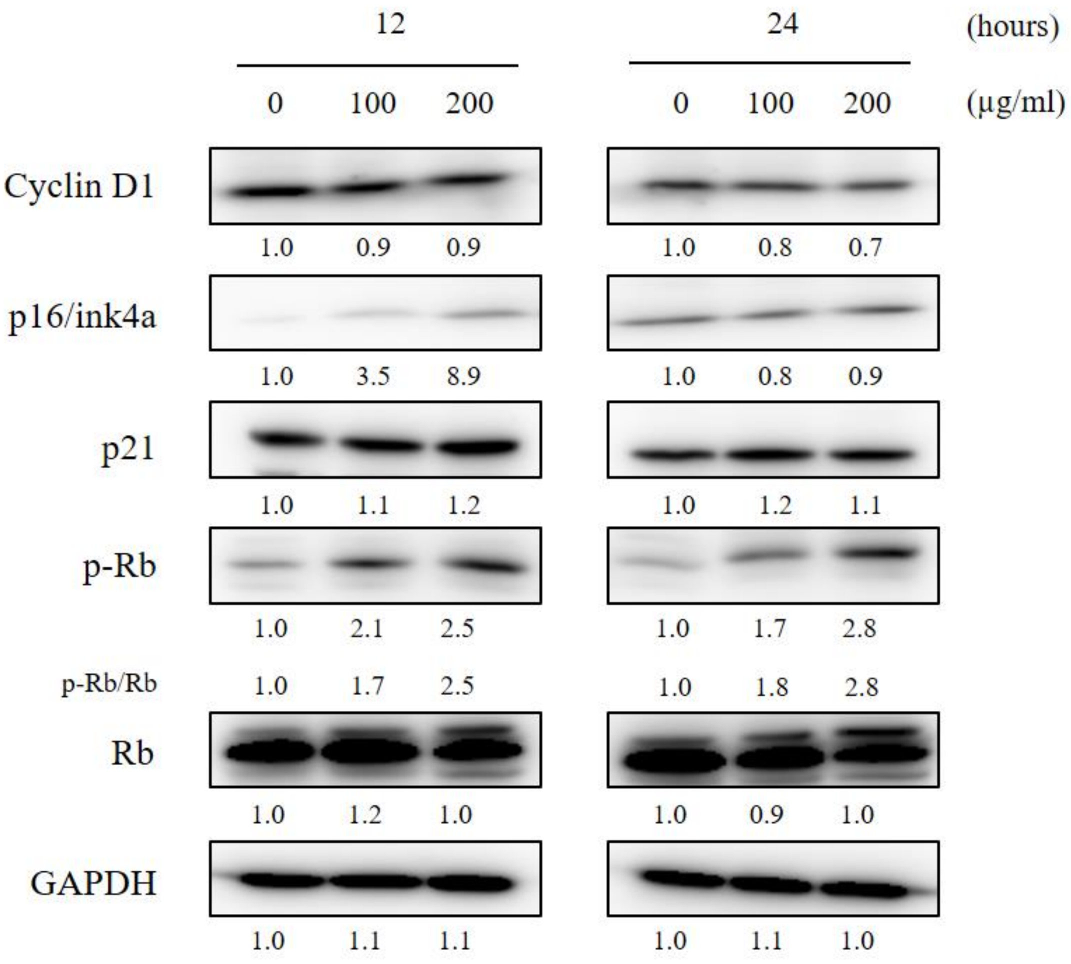

Figure S1. Induction of cell cycle arrest related proteins by *Mychonastes* sp. 246 methanolic extracts in BxPC-3 pancreatic cancer cells. BxPC-3 cells were treated with ME (0, 100, and 200 µg/mL) for 12 or 24 h and analyzed by Western blotting with Cyclin D1, p16/ink4a, p21, p-Rb, Rb and GAPDH.
